# Extrinsic Left Atrial Compression: An Echocardiography-Guided Diagnosis Illustrated by Two Clinical Cases and a Structured Review of Published Cases

**DOI:** 10.3390/jcm15072611

**Published:** 2026-03-29

**Authors:** Angelina Borizanova, Elena Kinova, Semra Beyti, Todor Angelov, Plamen Getsov, Assen Goudev

**Affiliations:** 1 Department of Emergency Medicine, Medical University Sofia, 1431 Sofia, Bulgaria; 2 Clinic of Cardiology, University Hospital “Tsaritsa Yoanna—ISUL”, 1527 Sofia, Bulgaria; 3 Department of Imaging Diagnostics, University Hospital “Tsaritsa Yoanna—ISUL”, Medical University Sofia, 1527 Sofia, Bulgaria; 4 Department of Gastroenterology, University Hospital “Tsaritsa Yoanna—ISUL”, Medical University Sofia, 1527 Sofia, Bulgaria

**Keywords:** left atrial compression, extrinsic cardiac compression, transthoracic echocardiography, hiatal hernia, mediastinal mass

## Abstract

**Background**: Extrinsic compression of the left atrium (LA) is a rare and underrecognized condition that may result in significant hemodynamic compromise and atrial arrhythmias. The available evidence has been largely limited to isolated case reports and small case series, and clinical awareness has remained low. **Methods**: We performed a structured review of published case reports and case series indexed in PubMed between 2016 and 2026 describing extracardiac LA compression. A predefined and reproducible literature search strategy with explicit eligibility criteria was applied. The structured review included 22 publications reporting 23 individual cases of LA compression; in addition, two institutional cases with distinct etiologies were presented separately. Demographic characteristics, presenting symptoms, diagnostic modalities, complications, management strategies, and outcomes were synthesized descriptively. **Results**: The structured review identified gastroesophageal disorders, particularly hiatal hernia, as the most frequent etiology, followed by vascular, mediastinal, malignant, and musculoskeletal causes. Dyspnea was the most common presenting symptom, while hemodynamic compromise, pulmonary edema, and atrial arrhythmia represented the most frequent complications. Transthoracic echocardiography was the initial diagnostic modality in all reported cases, with computed tomography required for definitive etiological diagnosis. The two institutional cases illustrated both a common cause, hiatal hernia mimicking intracardiac mass, and a rare, aggressive malignant cause with extensive mediastinal involvement. **Conclusions**: Extrinsic LA compression arises from diverse extracardiac pathologies and may be clinically severe. Transthoracic echocardiography can serve as a pivotal first-line tool for early recognition and differentiation from intracardiac masses, while cross-sectional imaging is essential for etiological clarification. By integrating institutional experience with a structured synthesis of published cases, this review can provide practical insights to support timely diagnosis and management of this potentially life-threatening condition.

## 1. Introduction

Extrinsic compression of the left atrium (LA) by extracardiac structures is most often detected as an incidental finding on imaging studies [1]. Although frequently asymptomatic, in rare cases it may lead to a broad spectrum of clinical manifestations, ranging from tachyarrhythmia and progressive dyspnea to pulmonary edema and hemodynamic compromise [2,3,4,5]. The susceptibility of the LA to external compression is related to its thin wall and low intraluminal pressure, which may result in impaired atrial filling, reduced cardiac output, and pulmonary venous hypertension [1]. In this context, transthoracic echocardiography (TTE) plays a pivotal role as a point-of-care imaging modality, allowing rapid bedside assessment and early identification of extrinsic causes of LA compression, particularly in emergency and acute care settings.

Given the rarity of reported cases and the heterogeneity of underlying etiologies, available evidence remains limited to isolated case reports and small case series. In this study, we present two institutional cases with different etiologies, complemented by a structured review of the literature, aiming to increase awareness of this underrecognized condition and to provide clinically relevant insights into its presentation, diagnostic approach, and management.

## 2. Materials and Methods

### 2.1. Literature Search and Study Selection

This study was designed as a structured narrative review combined with illustrative institutional cases, rather than a formal systematic review conducted under full PRISMA criteria. A structured literature search was conducted via PubMed to identify published case reports and case series describing LA compression. The search covered a ten-year period (2016–2026) and was performed using following Boolean string: “left atrial compression”, “extrinsic left atrial compression”, “atrial extrinsic compression”, “left atrial mass effect” and “case report” or “case series”. Only English-language articles were included, which may have introduced language bias. The search was limited to PubMed, which provided broad coverage of biomedical case reports and case series; however, this approach may have resulted in the omission of relevant cases indexed in other databases.

The initial search yielded 376 records. Titles and abstracts were screened individually in a structured manner for relevance, as the results included articles in which the search terms appeared separately and were not related to true LA compression. Studies were included if they reported original clinical cases (case reports or case series), describing the clinical presentation, diagnostic findings, management, or outcomes of LA compression by extracardiac structures. Review articles without primary patient data, animal studies, abstracts without full-text availability, duplicates, and unrelated publications were excluded.

After full-text evaluation, 22 publications met the inclusion criteria and were included in the final qualitative synthesis. A detailed summary of the published cases is provided in Table A1 [6,7,8,9,10,11,12,13,14,15,16,17,18,19,20,21,22,23,24,25,26]. This approach was consistent with prior narrative syntheses of rare clinical entities based on case-level evidence.

### 2.2. Data Extraction and Synthesis

For each eligible case, we extracted information on demographics, presenting symptoms, comorbidities, diagnostic methods, complications, treatment strategies, and patient outcomes. Data extraction was performed independently by two authors and cross-checked for accuracy. Any discrepancies were resolved through consensus discussion to ensure consistency and minimize subjective bias. Given the descriptive and heterogeneous nature of the included case reports, no formal risk-of-bias assessment tool was applied; however, potential sources of reporting bias (such as incomplete follow-up or missing data) were qualitatively considered during data synthesis. Due to the substantial heterogeneity in study design, patient characteristics, underlying etiologies, and outcome reporting, quantitative synthesis (meta-analysis) was not feasible; therefore, the findings were synthesized narratively. Results are presented through tables and figures to highlight recurrent clinical patterns. The synthesis aimed to summarize demographic characteristics, diagnostic approaches, management strategies, and outcomes. Given that the included evidence consisted exclusively of case reports and small case series, formal risk-of-bias assessment tools were not applicable. Instead, potential sources of bias, including selective reporting, publication bias, and incomplete follow-up, were considered qualitatively during data synthesis.

**Figure A1:**
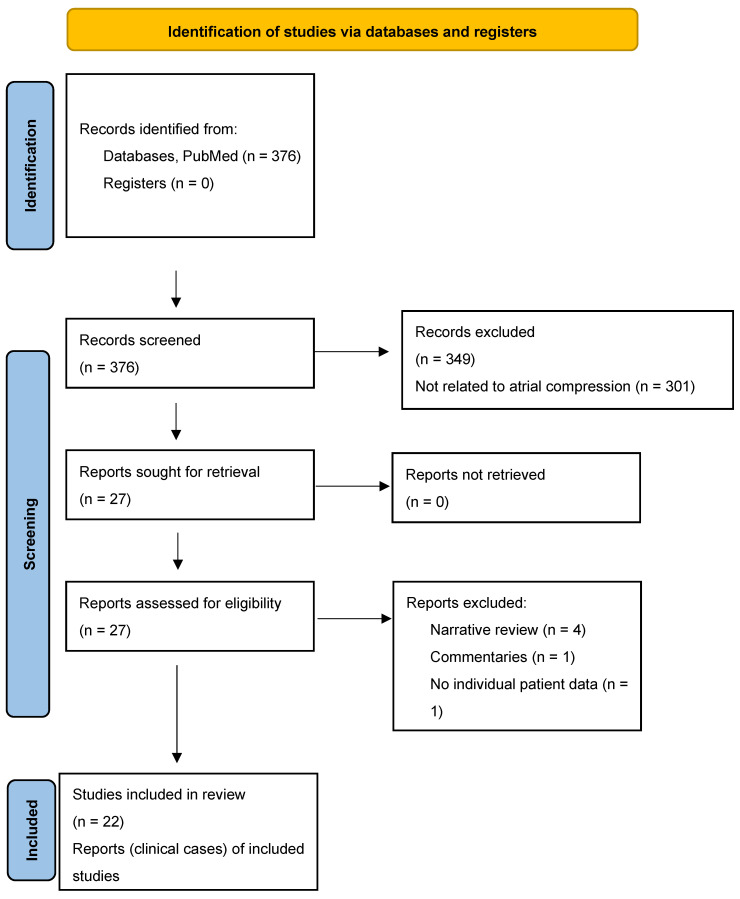
Flow diagram illustrates the literature search and study selection process.

The literature search and study selection process are illustrated in Figure A1 [27].

## 3. Results

This structured review identified 22 publications reporting 23 individual cases of LA compression between 2016 and 2026, as well as one article that described two distinct cases. Patient ages ranged from 22 to 89 years with a predominance of elderly individuals, and the majority of patients were female. The etiologies of LA compression were heterogeneous and could be grouped into several major categories. Gastroesophageal and foregut-related conditions represented the most frequent causes, including hiatal hernia, achalasia, esophageal distension or tamponade, atonic esophagus, esophageal stent placement, and foregut duplication cysts. Vascular causes constituted the second-most common group and included thoracic aortic aneurysms, dissecting aortic aneurysms, intramural aortic hematoma, ascending and descending aortic aneurysms, and pulmonary artery pseudoaneurysms. Less frequent etiologies comprised mediastinal masses, such as bronchogenic cysts and lung cancer, as well as musculoskeletal abnormalities, including vertebral column deformities and spinal osteophytes.

**Figure 1:**
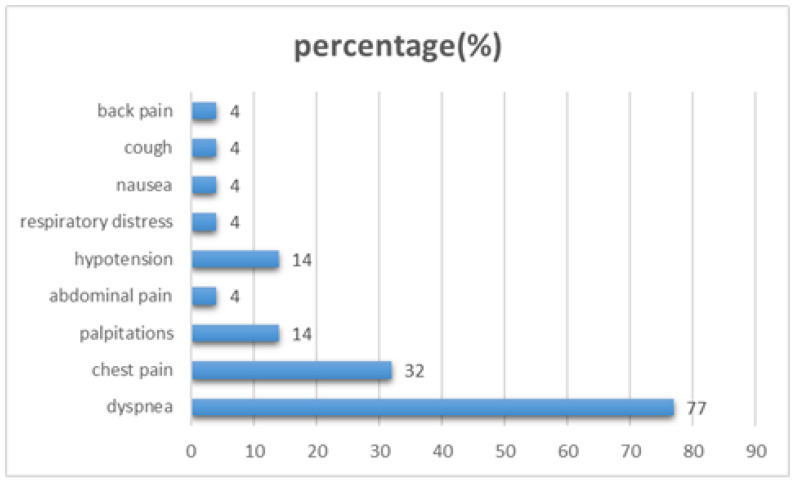
Spectrum of symptoms in patients with left atrial compression.

The most common presenting symptom was dyspnea, followed by chest pain, hypotension, and palpitations (Figure 1).

**Figure 2:**
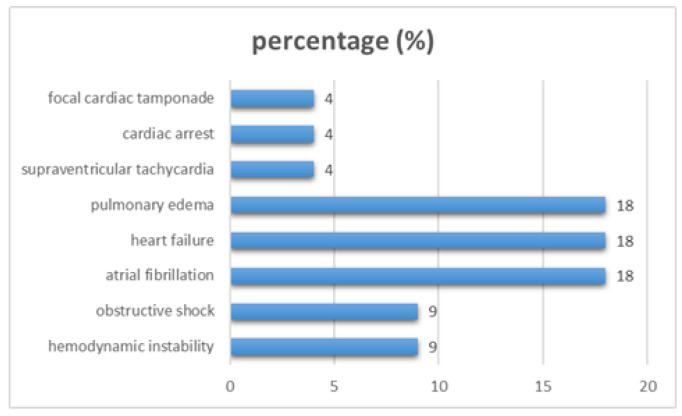
Complications that are associated with extrinsic left atrial compression.

In several cases, LA compression was associated with significant hemodynamic compromise, including obstructive shock, pulmonary edema, focal cardiac tamponade, and cardiac arrest. Supraventricular arrhythmias, particularly atrial fibrillation and atrial tachycardia, were reported in multiple cases, highlighting the potential arrhythmogenic impact of mechanical LA deformation (Figure 2).

### 3.1. Diagnostic Evaluation

Transthoracic echocardiography was used as the initial diagnostic modality in all reported cases, serving as a key screening tool by demonstrating LA compression or abnormal atrial geometry in a case-based clinical context. However, definitive etiological diagnosis required cross-sectional imaging, most commonly computed tomography, which identified the extracardiac structure responsible for compression. Additional diagnostic techniques, such as transesophageal echocardiography, endoscopy, or endobronchial ultrasound were applied selectively depending on the suspected underlying pathology.

### 3.2. Treatment and Outcomes

Management strategies varied accordingly to etiology and clinical severity. Surgical or interventional treatment was frequently employed for gastroesophageal and mediastinal causes, including hiatal hernia repair, cyst resection, and endovascular procedures, often resulting in clinical improvement or symptom resolution. In contrast, cases related to vascular pathology were associated with a higher rate of adverse outcomes, including refractory shock and mortality, particularly when definitive intervention was not feasible. Overall, patient outcomes ranged from full recovery to death, underscoring the potential severity of LA compression and the importance of early recognition and etiological treatment.

### 3.3. Case Presentation 1

A 41-year-old previously healthy man presented in 2017 with progressive chest tightness, dyspnea, and abdominal discomfort. On admission, he was hypotensive (90/60 mmHg) with sinus tachycardia but without shock or signs of peripheral hypoperfusion. Physical examination revealed hepatomegaly and a palpable abdominal mass. Laboratory tests showed mild renal impairment, mildly elevated inflammatory markers, and markedly increased D-dimer, while cardiac troponins were normal.

**Figure 3:**
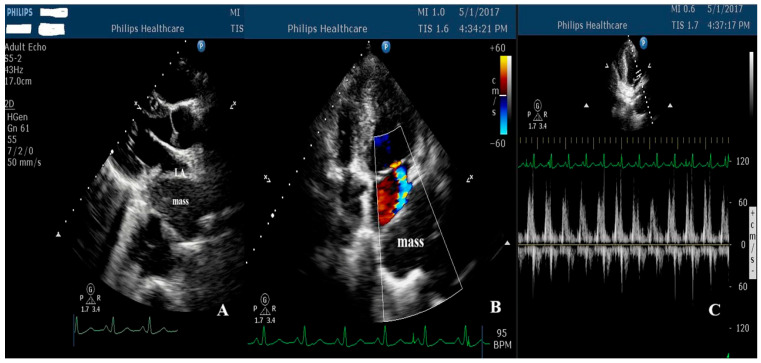
Transthoracic echocardiography showing hemodynamic consequences of an extracardiac mass. (**A**) Parasternal long-axis view showing the mass located posterior to the left atrium. (**B**) Apical four-chamber view with color Doppler imaging showing the mass adjacent to the left atrium, without evidence of internal vascular flow. (**C**) Pulsed-wave Doppler of the transmitral inflow demonstrating pronounced respiratory variations in flow velocities, reflecting functional compression of the left atrium.

Transthoracic echocardiography demonstrated severe extrinsic compression of the LA, with pronounced respiratory variations in transmitral inflow, consistent with hemodynamically significant LA compression, and preserved left ventricular systolic function (Figure 3).

**Figure 4:**
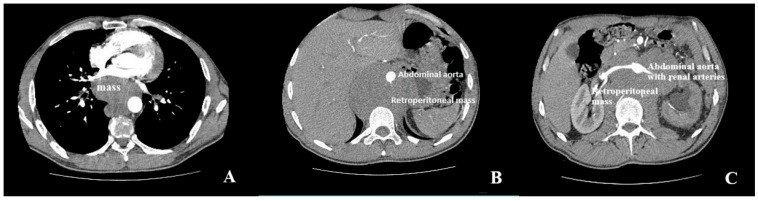
Contrast-enhanced computed tomography showing thoracic and retroperitoneal masses. (**A**) Axial contrast-enhanced CT showing a posterior mediastinal mass adjacent to the heart. (**B**) Axial abdominal CT showing a bulky retroperitoneal mass encasing the abdominal aorta, resulting in the “floating aorta” sign. (**C**) Axial abdominal CT showing a retroperitoneal mass encasing the abdominal aorta and renal vessels.

Contrast-enhanced CT revealed extensive confluent mediastinal and retroperitoneal lymphadenopathy causing marked LA compression, with circumferential encasement of the thoracic and abdominal aorta, as well as anterior displacement of the abdominal aorta and inferior vena cava (“floating aorta sign”) [28], without aortic wall invasion (Figure 4).

Despite these findings, the patient remained clinically stable and responded to initial conservative management; therefore, urgent surgical decompression was not pursued, given the diffuse extracardiac tumoral nature of the compression and the absence of a focal surgically accessible target.

A biopsy confirmed a non-seminomatous germ cell tumor; however, though systemic chemotherapy was initiated, the disease progressed aggressively and the patient died within one year of the diagnosis.

### 3.4. Case Presentation 2

**Figure 5:**
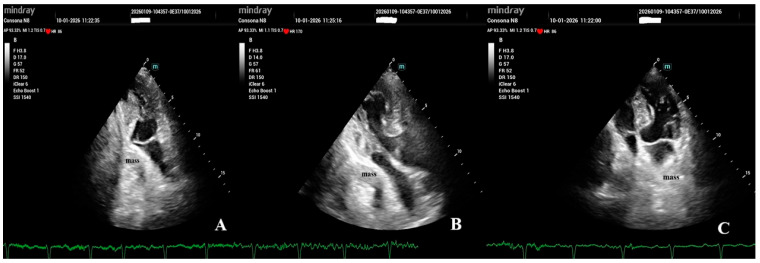
Transthoracic echocardiography showing a hiatal hernia, mimicking cardiac mass. (**A**) Apical two-chamber view showing a posteriorly located mass. (**B**) Apical three-chamber view showing the mass in a posterolateral position. (**C**) Apical four-chamber view showing a mass overlying the left atrium, consistent with a hiatal hernia.

An 84-year-old man was referred for pre-anesthetic cardiology evaluation prior to elective upper gastrointestinal endoscopy, indicated for mild anemia and progressive dysphagia. His medical history was notable for long-standing arterial hypertension. The patient reported intermittent palpitations, predominantly after meals, and a sensation of chest heaviness. On physical examination, he was normotensive with a normal heart rate. Cardiac auscultation revealed a holosystolic murmur, best heard at the apex with radiation to the left axilla. No signs of heart failure or peripheral congestion were present. Electrocardiography showed a sinus rhythm with first-degree AV block and frequent supraventricular premature beats. Transthoracic echocardiography demonstrated mild concentric left ventricular hypertrophy with preserved regional wall motion and normal left ventricular ejection fraction. Doppler analysis revealed grade 1 diastolic dysfunction. The mitral valve showed anterior leaflet prolapse (A2 scallop) with moderate mitral regurgitation. In the apical four-chamber view, a posterior echogenic structure was visualized projecting into the left atrial cavity, initially raising suspicions of an intracavitary LA mass; however, the LA endocardial borders remained smooth and intact and without evidence of attachment or independent mobility. In the apical three-chamber view, the structure was more clearly delineated as extracardiac, compressing the LA from a posterolateral direction, consistent with extrinsic LA compression (Figure 5). No significant respiratory variations in transmitral inflow velocities were observed.

**Figure 6:**
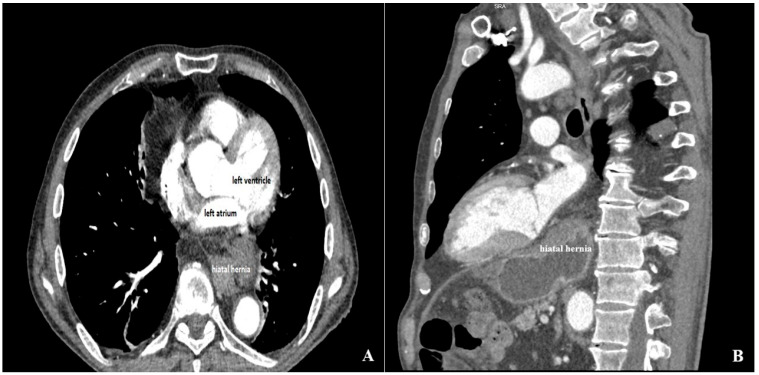
Contrast-enhanced computed tomography showing extracardiac origin. (**A**) Axial chest CT image showing a hiatal hernia located posterior to the left atrium. (**B**) Sagittal reconstruction showing posteriorly positioned herniation of the stomach through the esophageal hiatus, adjacent to the left atrium, which confirmed the extracardiac nature of the mass visualized on transthoracic echocardiography.

To further characterize this finding and differentiate it from an intracardiac mass, specific echocardiographic maneuvers suggestive of a hiatal hernia were systematically applied. The structure appeared at its maximal size when the LA was visualized in posterior imaging planes, while becoming smaller or absent in more anterior planes. The degree of LA encroachment varied dynamically with the respiratory cycle. In addition, partial loss of the normally sharply defined sonolucent contour of the descending thoracic aorta was noted due to superimposition of the extracardiac structure. Most importantly, following oral ingestion of a carbonated beverage, swirling echodensities were visualized within the structure, consistent with intraluminal air-fluid movement. Collectively, these findings strongly supported the diagnosis of a hiatal hernia causing extrinsic LA compression. Upper gastrointestinal endoscopy and contrast-enhanced computed tomography were then performed; consequently, as shown in Figure 6, these confirmed the presence of a hiatal hernia. Importantly, gastroscopy revealed esophageal carcinoma, which was identified as the primary cause of the patient’s dysphagia, anemia.

## 4. Discussion

This study integrates a ten-year structured review of published case reports and case series with two additional institutional cases, further expanding the limited body of case-level evidence on extrinsic LA compression. The findings supported the concept that LA compression represents a final common manifestation of a broad spectrum of extracardiac pathologies rather than a distinct disease entity.

Importantly, the two institutional cases presented in this study reflect and complement key patterns identified in the structured literature review. The first case illustrated a rare malignant etiology associated with aggressive clinical course and poor prognosis, consistent with previously reported tumor-related causes of LA compression. In contrast, the second case represented the most frequently observed mechanism identified in the review (hiatal hernia), while highlighting the diagnostic challenges and the pivotal role of transthoracic echocardiography in differentiating extracardiac compression from intracardiac masses. Together, these cases can provide real-world clinical validation of the heterogeneity of etiologies and the variable clinical impact observed across the reviewed literature.

The left atrium occupies a central anatomical position within the mediastinum and lies in close proximity to the esophagus, descending thoracic aorta, pulmonary veins, and surrounding mediastinal structures [1,15]. A wide range of extracardiac conditions may therefore result in LA compression, including distended gastrointestinal structures, mediastinal masses, intrapericardial and aortic abnormalities, and pulmonary lesions [5,6,7,8,9,10,11,12,13,14,15,16,17,18,19,20]. The susceptibility of the LA to external compression is related to its thin wall and relatively low intraluminal pressure, which may result in impaired atrial filling, reduced cardiac output, and pulmonary venous hypertension. Consequently, extrinsic LA compression may mimic more common cardiac, pulmonary, or gastrointestinal disorders, leading to delayed diagnosis and, in some cases, life-threatening outcome [14,15,16,17,18,19,20,21,22,23,24,25].

In line with previously published case-based reports, gastroesophageal disorders (particularly hiatal hernia) have emerged as the most frequently reported cause of LA compression, reflecting the close anatomical relationship between the posterior LA wall and the esophagus (2,3,5,6). Our second case illustrated this common etiology while emphasizing the diagnostic value of TTE in differentiating extrinsic compression from true intracavitary LA masses. Dynamic changes across imaging planes, respiratory variability, loss of the normal descending aortic contour, and the appearance of swirling echodensities after ingestion of a carbonated beverage can represent simple but highly suggestive echocardiographic features that may guide early etiological suspicions [29]. Importantly, this case highlighted the need for a structured diagnostic approach, as symptoms that were initially attributable to a seemingly benign hiatal hernia ultimately led to the detection of an underlying esophageal carcinoma.

Conversely, the first institutional case highlighted a rare and aggressive mechanism of LA compression caused by an extragonadal non-seminomatous germ cell tumor, resulting in extensive mediastinal infiltration and circumferential aortic encasement. Tumor-related LA compression has remained uncommon and has been associated with limited therapeutic options and poor prognosis [26], particularly when compression has arisen from diffuse, non-resectable disease rather than focal mass effect.

Across the reviewed cases, dyspnea was the most frequently reported presenting symptom, followed by chest pain, hypotension, and palpitations [4,5,6,7,8,9,10,11,12,13,14,15,16,17,18,19,20,21,22,23,24,25,26]. The predominance of dyspnea can be pathophysiologically plausible, as even modest elevation in LA pressure, secondary to external compression, are directly transmitted to the pulmonary venous circulation, and can lead to pulmonary congestion, reduced lung compliance, and impaired gas exchange. Furthermore, restriction of LA filling may reduce left ventricular preload and stroke volume, thereby contributing to exertional intolerance and progressive shortness of breath [11,20,26].

Several reports described severe complications, including hemodynamic instability, obstructive shock, pulmonary edema, atrial arrhythmias, and cardiac arrest [5,6,7,8,9,10,11,12,13,14,15,16,17,18,19,20,21,22,23,24,25,26], underscoring the potential for rapid clinical deterioration in selected patients. In cases of marked compression, substantial limitation of ventricular filling may critically reduce cardiac output. When compensatory neurohumoral mechanisms fail, this hemodynamic compromise may culminate in hypotension and, in extreme scenarios, obstructive shock, resembling the physiology of cardiac tamponade or severe mitral inflow obstruction. In addition to hemodynamic consequences, mechanical deformation of the atrial wall may promote electrical instability by altering atrial stretch and conduction properties, thereby becoming predisposed to atrial arrhythmias [3,4,15,21]. Collectively, these observations have reinforced the concept that extrinsic LA compression is not merely an imaging finding but a potentially dynamic and clinically significant condition.

Transthoracic echocardiography served as the initial diagnostic modality in all reported cases [4,5,6,7,8,9,10,11,12,13,14,15,16,17,18,19,20,21,22,23,24,25,26], underscoring its pivotal role in the early recognition of extracardiac structures mimicking intracardiac pathology, particularly in acute and emergency settings. Beyond its wide availability and bedside applicability, TTE can enable real-time anatomical and functional assessment through a systemic multiplanar approach. Careful interrogation of the parasternal long axis, apical four-chamber, two-chamber, and three-chamber views are essential for accurate delineation of the suspected mass and its spatial relationship to the LA.

Posterior extracardiac structures, such as a hiatal hernia, are typically visualized as echodense heterogeneous masses adjacent to or overlying the LA, often best appreciated in apical and parasternal projections. Certain echocardiographic features may serve as “red flags” that suggest an extracardiac origin [19,29]. These include the absence of a clear attachment to the atrial wall or interatrial septum, a heterogeneous appearance with internal echolucent areas, and subtle internal swirling echoes corresponding to intraluminal gastric contents. Dynamic changes related to respiration or patient positioning, which are atypical for true intracardiac tumors, can further support these suspicions [29].

Doppler interrogation added a crucial hemodynamic dimension to the evaluation. Pulsed-wave Doppler assessment of the transmitral inflow may demonstrate exaggerated respiratory variation or impaired diastolic filling in cases of significant LA compression. In more advanced scenarios, reduced left ventricular outflow tract velocity time integral and decreased calculated stroke volume may indicate compromised preload and diminished cardiac output [30]. These parameters can allow differentiation between an incidental anatomical impression and clinically relevant hemodynamic compromise.

Furthermore, the institutional cases reinforced the central role of echocardiography as an initial diagnostic tool, as observed across all the reviewed cases. In both patients, transthoracic echocardiography enabled early recognition of LA deformation and guided further diagnostic evaluation. The dynamic echocardiographic features observed in the second case, including the respiratory variability and intraluminal swirling echoes following ingestion of a carbonated beverage, further illustrated the practical bedside findings that may support differentiation from intracardiac pathology. These observations aligned closely with patterns identified in the literature and emphasized the importance of systematic echocardiographic assessment.

Despite its diagnostic strength, definitive etiological characterization consistently required cross-sectional imaging, most commonly computed tomography, which precisely defined the extracardiac origin, anatomical relationships, and extent of compression. Clinical management and outcomes were largely determined by the underlying cause, as gastroesophageal and focal mediastinal etiologies were frequently associated with favorable outcomes following definitive intervention, while vascular and malignant causes carried substantially higher morbidity and mortality [4,5,6,7,8,9,10,11,12,13,14,15,16,17,18,19,20,21,22,23,24,25,26].

By integrating institutional experience with a structured synthesis of published cases, this study bridged the gap between isolated case reports and clinically applicable insights, providing a more comprehensive understanding of this rare but potentially life-threatening condition.

### 4.1. Future Perspectives and Research Directions

Despite the growing number of published case reports, extrinsic LA compression has remained an underrecognized and poorly characterized clinical entity. The current evidence base is almost exclusively derived from isolated case reports and small case series, underscoring the need for more systematic data collection. The establishment of multi-center registries dedicated to extracardiac cardiac compression syndromes would allow for better characterizations of epidemiology, etiological patterns, clinical trajectories, and outcomes.

Another major unmet need is the development of standardized echocardiographic diagnostic criteria. At present, the identification of LA compression has relied on qualitative assessment and operator experience. Future studies should aim to define reproducible echocardiographic parameters, including thresholds for respiratory variations in transmitral inflow, degrees of atrial deformation, and quantitative indices of left ventricular outflow tract velocity time integral reduction. Such criteria could improve diagnostic consistency and facilitate earlier recognition of hemodynamically significant cases.

Prospective observational studies are also warranted to clarify the natural history of both acute and chronic forms of LA compression. Key questions remain regarding the reversibility of structural and functional alterations, the incidence of atrial arrhythmias, and the predictors of clinical deterioration. Identifying hemodynamic thresholds that warrant urgent intervention could represent another important research priority, particularly in patients presenting with hypotension or early signs of obstructive physiology.

Ultimately, the integration of multimodality imaging with standardized hemodynamic assessments may allow the development of a structured diagnostic and therapeutic algorithm. Greater awareness and collaborative research efforts will be essential to move beyond descriptive case-level evidence toward evidence-based management strategies for this potentially life-threatening condition.

### 4.2. Study Limitations

This study was limited by its reliance on published case reports and small case series, which were inherently subject to publication and reporting bias, and preclude an estimation of true incidence or prognosis. The descriptive and heterogeneous nature of the available data prevented formal risk-of-bias assessments, meta-analyses, and consistent comparisons across cases. In addition, the restriction to PubMed and English-language publications may have resulted in an incomplete case capture. Finally, the two institutional cases, although illustrative, represented a limited sample and may not be generalizable.

## 5. Conclusions

Extrinsic compression of the LA is an underrecognized but potentially life-threatening condition, arising from a wide spectrum of extracardiac pathologies. Transthoracic echocardiography serves as a pivotal first-line tool for early recognition and differentiation from intracardiac masses, while definitive etiological diagnosis requires cross-sectional imaging. Increased awareness of this entity and its diverse causes is essential, as timely recognition and etiology-directed management may be lifesaving.

## Data Availability

No new datasets were generated for this study. Data extracted from published case reports are available within the article. Clinical data from the institutional cases are not publicly available due to patient privacy and ethical restrictions.

## Abbreviations

The following abbreviations are used in this manuscript:

LA	Left Atrium
TTE	Transthoracic Echocardiography
CT	Computed Tomography
AV	Atrio-Ventricular

## Appendix A

### Appendix A.1

Table A1 presents the characteristics of published case reports and case series included in the structured literature review.

**Table A1:** Published case reports describing left atrial compression.

Journal	Year	Age/Sex	Etiology	Symptoms	Complications	Primary Diagnostics	Additional Diagnostic	Treatment	Outcome
Cureus [4]	2024	85 y, female	Aortic intramural hematoma	Dyspnea, hypotension	Obstructive shock	Echo	CT	TEVAR, surgery	Death
The American Journal of Emergency Medicine [6]	2021	57 y, female	Hiatal hernia	Dyspnea	Heart failure	Echo	CT	Surgical HH repair	Discharged
CASE (Phila) [7]	2022	50 y, female	Focal (posterior) hemopericardium	Abdominal pain, nausea, dyspnea	Focal cardiac tamponade	Echo	CT	Cardiothoracic surgery	Discharged
Fed Pract [8]	2020	57 y, male	Infected bronchogenic cyst	Chest pain, dyspnea	None reported	CT	EBUS	EBUS-guided transbronchial needle aspiration, antibiotics	Discharged
Echocardiography [9]	2019	83 y, male	Angioedema, swollen esophagus	Respiratory distress, hypotension	Hemodynamic instability	Echo	CT	Corticosteroids, vasopressors, fluids	Death
HeartRhythm Case Reports [10]	2021	36 y, male	Esophageal stent	Palpitations, chest pain	Atrial fibrillation, atrial tachycardia	Echo	CT	Surgery	Unknown
Journal of Clinical Ultrasound [11]	2018	74 y, female	Atonic esophagus, degenerative neuropathy	Dyspnea, cough	Pulmonary edema	Echo	CT	Esophageal endoscopy, mechanical fragmentation of solid and compact bolus	Improved
CASE (Phila) [12]	2018	22 y, male	Achalasia	Dyspnea, cough	Pulmonary edema	Echo	CT	LES pneumatic endoscopic dilatation	Improved
Clin Pract Cases Emerg Med [13]	2025	64 y, female	Esophageal tamponade (esophageal and gastric distention)	Tachypnea, hypotension	Obstructive shock	Echo	CT	Nasogastric tube	Improved
BMJ Case Reports [14]	2017	35 y, male	Foregut duplication cyst (mediastinal cyst)	Palpitations, dyspnea	Supraventricular tachycardia	Echo	CT	Surgical resection	Improved
J Atr Fibrillation [15]	2016	66 y, female	Small cell lung cancer	Dyspnea, chest pain	Atrial fibrillation	Echo	CT	Chemotherapy	Improved
Echocardiography [16]	2020	43 y, female	Localized anterior bending of vertebral column between fifth and eighth thoracic vertebrae	Dyspnea, backpain	Pulmonary edema	Echo	CT	No treatment	Death
Balkan Med J [17]	2018	75 y, male	Dissecting aortic aneurysm	Chest pain, dyspnea	Cardiac arrest	Echo	CT	No treatment	Death
Lancet [18]	2018	76 y, female	Spinal osteophytes	Mid-diastolic murmur	Heart failure	Echo	CT	Orthopedic surgery	Improved
Eur J Case Rep Intern Med [19]	2023	68 y, female 78 y, female	Hiatal hernia Hiatal hernia	Chest pain, dyspnea, nausea Chest pain, dyspnea	Heart failure	Echo Echo	CT CT	Laparoscopic repair No treatment	Improved Death
Braz J Cardiovasc Surg. [20]	2020	89 y, female	Ascending and descending aortic aneurysms	Dyspnea, back pain	None reported	Echo	CT	No treatment	Unknown
Eur Heart J Case Rep [21]	2020	83 y, male	Hiatal hernia type 4	Dyspnea, hypotension	Hemodynamic instability	Echo	CT	Surgical treatment	Improvement
Rev Port Cardiol [22]	2018	80 y, female	Thoracic aortic aneurysm	Dyspnea, orthopnea, dry cough	Pulmonary edema, atrial fibrillation	Echo	CT	Medical therapy for symptom relief	Unknown
JACC Case Rep [23]	2020	40 y, female	Giant right pulmonary artery pseudoaneurysm	Chest pain	None reported	Echo	CT	Endovascular approach, stent implantation	Improved
J Tehran Heart Cent [24]	2019	82 y, male	Hiatal hernia	Dyspnea, palpitations	Atrial fibrillation	Echo	CT	Medical therapy	Follow-up
Anatol J Cardiol [25]	2021	73 y, man	Bronchogenic cyst	Chest pain	None reported	Echo	CT	Surgery	Death
Internal Medicine [26]	2009	21 y, male	Mediastinal testicular tumor	Dyspnea	Heart failure	Echo	CT	Chemotherapy	Referred for surgery, unknown outcome

Abbreviations: Echo—echocardiography, CT—computed tomography, y—years.
